# Impact of Cerebrospinal Fluid Shunting for Idiopathic Normal Pressure Hydrocephalus on the Amyloid Cascade

**DOI:** 10.1371/journal.pone.0119973

**Published:** 2015-03-30

**Authors:** Masao Moriya, Masakazu Miyajima, Madoka Nakajima, Ikuko Ogino, Hajime Arai

**Affiliations:** Department of Neurosurgery, Juntendo University Graduate School of medicine, Tokyo, Japan; UW Medicine Neuropathology, UNITED STATES

## Abstract

The aim of this study was to determine whether the improvement of cerebrospinal fluid (CSF) flow dynamics by CSF shunting, can suppress the oligomerization of amyloid β-peptide (Aβ), by measuring the levels of Alzheimer’s disease (AD)-related proteins in the CSF before and after lumboperitoneal shunting. Lumbar CSF from 32 patients with idiopathic normal pressure hydrocephalus (iNPH) (samples were obtained before and 1 year after shunting), 15 patients with AD, and 12 normal controls was analyzed for AD-related proteins and APLP1-derived Aβ-like peptides (APL1β) (a surrogate marker for Aβ). We found that before shunting, individuals with iNPH had significantly lower levels of soluble amyloid precursor proteins (sAPP) and Aβ38 compared to patients with AD and normal controls. We divided the patients with iNPH into patients with favorable (improvement ≥ 1 on the modified Rankin Scale) and unfavorable (no improvement on the modified Rankin Scale) outcomes. Compared to the unfavorable outcome group, the favorable outcome group showed significant increases in Aβ38, 40, 42, and phosphorylated-tau levels after shunting. In contrast, there were no significant changes in the levels of APL1β25, 27, and 28 after shunting. After shunting, we observed positive correlations between sAPPα and sAPPβ, Aβ38 and 42, and APL1β25 and 28, with shifts from sAPPβ to sAPPα, from APL1β28 to 25, and from Aβ42 to 38 in all patients with iNPH. Our results suggest that Aβ production remained unchanged by the shunt procedure because the levels of sAPP and APL1β were unchanged. Moreover, the shift of Aβ from oligomer to monomer due to the shift of Aβ42 (easy to aggregate) to Aβ38 (difficult to aggregate), and the improvement of interstitial-fluid flow, could lead to increased Aβ levels in the CSF. Our findings suggest that the shunting procedure can delay intracerebral deposition of Aβ in patients with iNPH.

## Introduction

Alzheimer’s disease (AD) is a neurodegenerative disorder and the most common form of dementia. In the brains of patients with AD, a toxic oligomeric species of the amyloid β-peptide (Aβ) induces synaptic degeneration and neuronal death. The amyloid cascade hypothesis posits that polymerization of Aβ and subsequent accumulation of this toxic species is the principal cause of AD pathogenesis [1, 2].

Another type of dementia, the normal pressure hydrocephalus (NPH), has been linked to the reduction in compliance between the cerebrospinal compartments and disruption in cerebrospinal fluid (CSF) outflow-absorption [3]. Idiopathic normal pressure hydrocephalus (iNPH), a disease of uncertain etiology affecting the elderly, causes gait disturbance, dementia, and urinary incontinence [4]. Shunting surgeries have been shown to be effective in more than 80% of patients with iNPH [5–7], and this is thought to be due to the relief of intracranial pressure caused by CSF accumulation. However, the mechanism by which shunt surgery improves the symptoms of iNPH is unclear. In patients with iNPH, the turnover of CSF appears to decline due to reduced CSF absorption. Treatment using CSF shunting not only corrects intracranial pressure but also effectively promotes the turnover of CSF, thus compensating for the decrease in CSF absorption caused by iNPH [8].

Interestingly, some studies have reported cases of comorbid iNPH and AD [9, 10], with a general decline in CSF turnover [11]. The production and turnover of CSF helps clear toxic molecules such as Aβ from the interstitial space in the brain to the bloodstream and lymphatic system [12, 13]. In AD, increased deposition of Aβ in the meninges leads to a greater resistance in CSF outflow. In iNPH, increased CSF pressure causes low CSF production and less clearance of Aβ. Failure of the CSF to clear toxic metabolites leads to the accumulation of Aβ in the brain of patients with AD and NPH [11]. CSF shunt surgery, typically performed to treat NPH, could promote CSF drainage and turnover, ultimately resulting in increased Aβ clearance [14].

Amyloid precursor protein (APP) plays a significant role in AD pathogenesis since its cleavage by the proteolytic enzymes, β- and γ-secretase, generates the various types of Aβ peptides. Of these, Aβ42 is a major component of senile plaques in patients with AD. APLP1-derived Aβ-like peptides (APL1β), homologues of APP, are similar to soluble APP (sAPP) β in its primary sequence and function [15]. However, APL1β does not aggregate and accumulate in the brain. Moreover, due to the oligomer formation, the level of Aβ42 in the CSF is not reflective of its production in the brain since it is difficult to directly measure small amounts of oligomers [16, 17]. Interestingly, most γ-secretase modulators that upregulate the relative production of Aβ42 cause a parallel increase in the production of APL1β28 in cultured cells [16]. Therefore, APL1β28 can be measured as a surrogate marker for Aβ42 production in the brain.

We hypothesized that the promotion of CSF production and drainage by CSF shunt surgery may suppress the oligomerization of Aβ and result in increased Aβ clearance. In the current study, in order to determine the effects of CSF shunting on the amyloid cascade, we measured the levels of AD-related proteins in the CSF of patients with iNPH, before and after the lumboperitoneal shunting (LPS) surgery. We also investigated the influence of oligomerization of Aβ by comparing the levels of Aβ42 in the CSF, and determining the changes in the production of Aβ42, as estimated by the levels of APL1β28.

## Materials and Methods

### Patients

LPS was performed on 32 patients with iNPH, which included 23 men and 9 women aged 73.7 ± 6.8 years (mean ± SD), between 2007 and 2012. Diagnostic criteria were symptoms and signs of iNPH in accordance with the Japanese guidelines for iNPH [2], and patients with secondary NPH were not included in this study. Score for the iNPH Grading Scale (iNPHGS) [18], mini mental state examination (MMSE), frontal assessment battery (FAB), Trail Making Test Part A (TMT-A), and modified Rankin Scale (mRS) [19, 20] were evaluated before LPS and 1 year after LPS. CSF was also sampled at the same times. The study design was approved by the Ethics Committee of Juntendo University, Japan. Written informed consent was obtained from patients and families prior to shunt placement for all patients who were positive for the tap test, which is a diagnostic tool used for selecting patients with iNPH for shunt surgery. In all patients, LPS was performed using adjustable valves (non-siphon control (NSC) valve with small lumen catheter ©Medtronic Neurosurgery, Goleta, CA).

Fifteen patients with AD, 11 men and 4 women, aged 71.5 ± 10.6 years (mean ± SD), were recruited in this study. AD was diagnosed using standard clinical criteria [21, 22].

Finally, 12 normal controls (NCs), 3 men and 9 women, aged 67.1 ± 11.0 years (mean ± SD), were recruited in this study. The NC group had no history of dementia and did not show any signs of other psychiatric illnesses. All patients with AD and NCs consented to lumbar punctures at the Juntendo University Hospital.

### CSF samples and biomarker assay

Lumbar puncture was performed in the L3-L4 or L4-L5 interspace before LPS. CSF was sampled through direct lumbar puncture before LPS, and through a puncture of the reservoir 1 year after LPS. All CSF samples were centrifuged at 3,000 rpm for 10 min at 4°C to remove cells and debris. Samples were aliquoted and stored in polypropylene tubes at −80°C until biochemical analyses. Levels of the CSF biomarkers, tau and phosphorylated tau (p-tau; at threonine 181) were determined using standardized, commercially available ELISA kits (Innotest hTau-Ag and Innotest Phosphotau (181P), Innogenetics, Ghent, Belgium). Levels of sAPPα and sAPPβ; Aβ38, 40, 42, and 43; and APL1β25, 27, and 28 were measured using specific ELISA kits obtained from Immuno Biological Laboratories (IBL, Gunma, Japan).

### Statistics

Non-parametric statistical methods were used in all analyses. The Wilcoxon signed-ranks test was used for within-group comparisons of mRS, iNPHGS, MMSE, FAB, and TMT-A scores, while the Mann-Whitney U test was used for comparisons between groups. The Spearman rank correlation coefficient (r) was used to estimate associations between variables. Statistical analyses were performed with IBM SPSS Version 18.0 (SPSS, Cary, NC, USA) for Windows, and p < 0.05, determined with a *t*-test, was considered significant.

## Results

### Clinical outcomes

**Fig 1:**
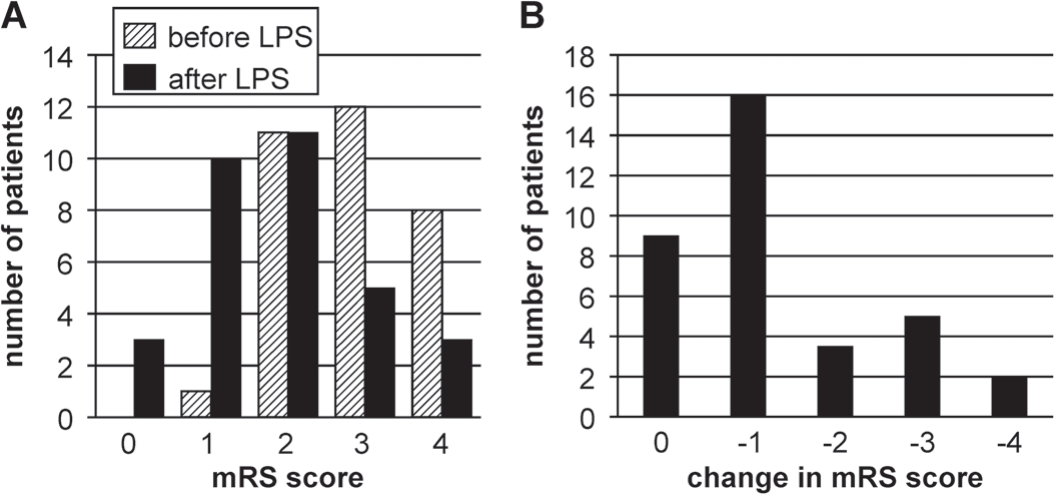
Bar charts of patient functional status based on their mRS score. A: Distribution of patients across mRS scores before and after LPS. B: Change in the median mRS scores 1 year after LPS. Abbreviations: LPS, lumboperitoneal shunting; mRS, modified Rankin Scale.

In 72% of the iNPH cases (23/32), the median mRS score improved by 1 point after LPS (Fig. 1A & B). Additionally, iNPHGS, MMSE, FAB, and TMT-A scores significantly improved 1 year after LPS (Table 1). We then divided the patients with iNPH into two groups: the favorable outcome group (23 patients: improvement ≥ 1 on mRS) and the unfavorable outcome group (9 patients: no improvement on mRS). In the favorable outcome group, iNPHGS total, MMSE, FAB, and TMT-A scores improved significantly. Even in the subgroup analysis with an unfavorable outcome, a significant improvement was noted in the TMT-A score with time.

**Table 1:** Comparison of iNPHGS scores and cognitive functions of patients with iNPH before and 1 year after LPS.

		Before	After	p value
iNPHGS total score	All patients (n = 32)	5 (4–7)	3 (1–6)	< 0.001
	Favorable outcome (n = 23)	5 (4–7)	2 (1–4)	< 0.001
	Unfavorable outcome (n = 9)	5 (3–9)	5 (3–8)	NS
MMSE	All patients (n = 32)	24 (21–27)	27 (22–29)	0.001
	Favorable outcome (n = 23)	24 (22–26)	27 (24–29)	0.002
	Unfavorable outcome (n = 9)	25 (19–27)	26 (18–27)	NS
FAB	All patients (n = 32)	13 (11–14)	15 (12–16)	0.021
	Favorable outcome (n = 23)	13 (12–14)	15 (12–16)	0.007
	Unfavorable outcome (n = 9)	14 (11–16)	14 (11–16)	NS
TMT-A	All patients (n = 32)	84 (61–106)	64 (46–84)	0.001
	Favorable outcome (n = 23)	79 (61–102)	68 (47–84)	0.005
	Unfavorable outcome (n = 9)	95 (56–122)	55 (46–89)	0.043

Abbreviations: before, before lumboperitoneal shunt; after, 1 year after lumboperitoneal shunt; FAB, frontal assessment battery; iNPH, idiopathic normal pressure hydrocephalus; iNPHGS, iNPH grading Scale; LPS, lumboperitoneal shunt; MMSE, Mini Mental State Examination; NS, non-significant; TMT-A, Trail Making Test Part A

Data are medians. All *P* values were obtained using the Wilcoxon test.

### CSF analyses

Comparisons of CSF measurements indicated that the levels of sAPP, sAPPα, sAPPβ, and Aβ38 were not significantly different between the AD and NC groups (Table 2).

**Table 2:** Comparison of CSF values between groups.

	iNPH (n = 32)(before LPS)	AD (n = 15)	NC (n = 12)	p1, p2
**sAPP *(ng/mL)***	478 (244)	1274 (216)	1064 (181)	p1 < 0.001, p2 < 0.001
**sAPPα *(ng/mL)***	137 (64)	358 (84)	343 (89)	p1 < 0.001, p2 < 0.001
**sAPPβ *(ng/mL)***	168 (92)	373 (129)	317 (60)	p1 < 0.001, p2 < 0.001
**Aβ38 *(pg/mL)***	1469 (1007)	2963 (1596)	2965 (1251)	p1 = 0.030, p2 = 0.010
**Aβ40 *(pg/mL)***	7530 (5581)	10777 (8457)	14088 (10079)	p1 = NS, p2 = NS
**Aβ42 *(pg/mL)***	241 (195)	73.0 (62)	293 (111)	p1 < 0.001, p2 = NS
**Aβ43 *(pg/mL)***	34.6 (44.8)	15.4 (16.1)	14.2 (7.3)	p1 = NS, p2 = NS
**APL1β25 *(pg/mL)***	2188 (444)	2322 (549)	2459 (943)	p1 = NS, p2 = NS
**APL1β27 *(pg/mL)***	707 (188)	1076 (671)	669 (172)	p1 = NS, p2 = NS
**APL1β28 *(pg/mL)***	1091 (318)	1153 (295)	1092 (294)	p1 = NS, p2 = NS
**Tau *(pg/mL)***	112 (96)	533 (383)	203 (114)	p1 = 0.002, p2 = NS
**p-tau *(pg/mL)***	19.2 (6.5)	79.7 (47.7)	31.5 (9.7)	p1 < 0.001, p2 = 0.004
**Protein *(pg/mL)***	34.4 (9.2)	37.9 (9.1)	35.3 (8.0)	p1 = NS, p2 = NS
**Aβ42/p-tau**	19.7 (20.3)	5.3 (3.7)	10.8 (5.4)	p1 = 0.01, p2 = NS

Abbreviations: Aβ, amyloid β-peptide; AD, Alzheimer’s disease; APL1β, APLP1-derived Aβ like peptide; CSF, cerebrospinal fluid; iNPH, idiopathic normal pressure hydrocephalus; NC, normal control; NS, non-significant; p-tau, tau phosphorylated at threonine 181; sAPP, soluble amyloid precursor protein

p1, comparison between iNPH and AD; p2, comparison between iNPH and NC.

All *P* values were obtained using the Wilcoxon test. SD values are given in parentheses.

Correlation is significant at the 0.05 level.

However, their levels were significantly lower in patients with iNPH before LPS than in patients with AD and NCs. Aβ42 level and Aβ42/p-tau ratio were significantly lower in patients with AD than in individuals with NC and iNPH, before LPS. The CSF levels of tau and p-tau were significantly higher in patients with AD than in NCs and patients with iNPH before LPS.

**Fig 2:**
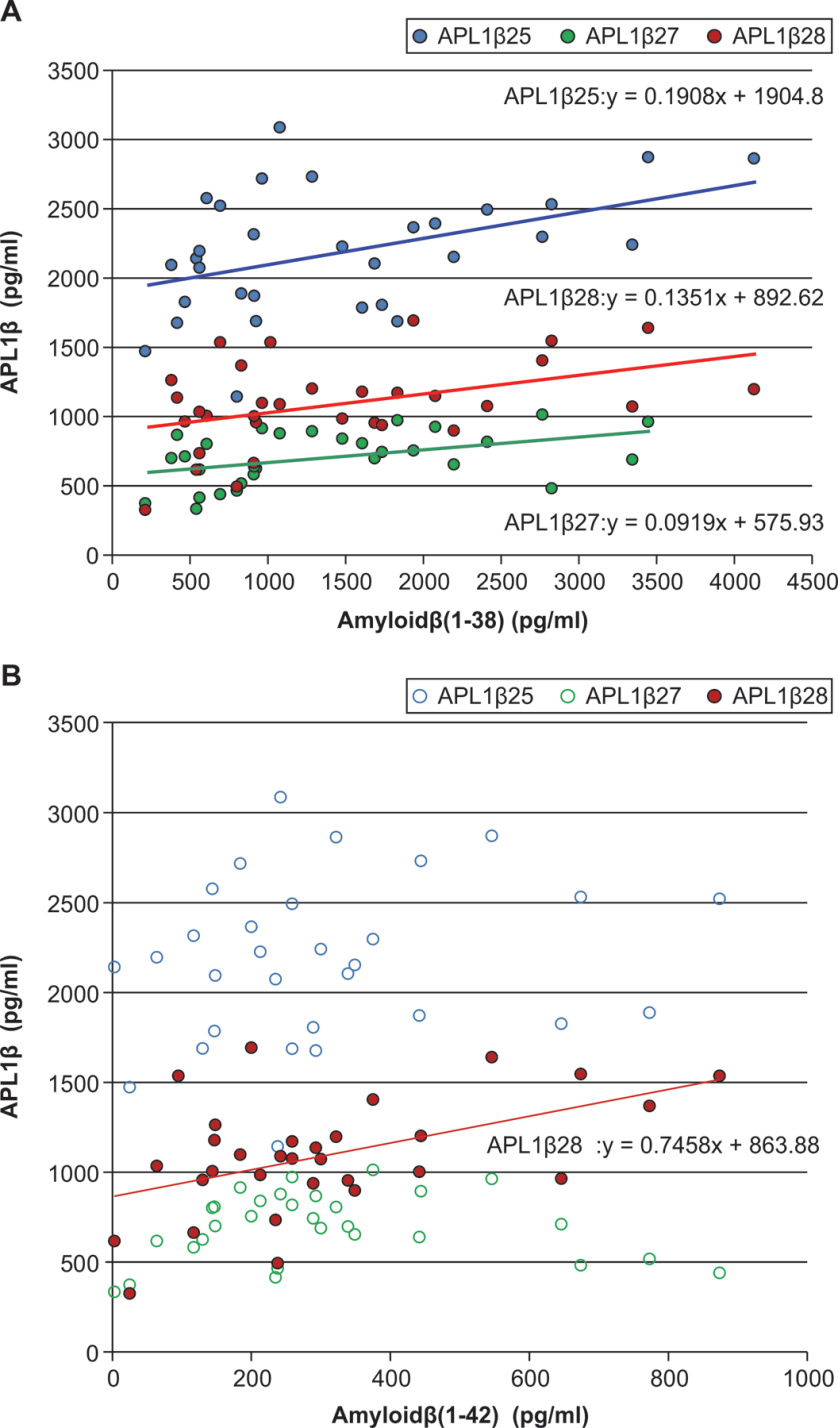
Correlation between Aβ38, 40 levels, and APL1β (25, 27, and 28) in patients with iNPH before LPS. A: Association between APL1β (25, 27, and 28) and Aβ38. B: Association between APL1β (25, 27, and 28) and Aβ42. Abbreviations: Aβ, amyloid β-peptide; APL1β, APLP1-derived Aβ like peptide; iNPH, idiopathic normal pressure hydrocephalus; LPS, lumboperitoneal shunting.

We found positive correlations between Aβ38 levels and APL1β25 (r = 0.467, p = 0.008), APL1β27 (r = 0.482, p = 0.006), and APL1β28 (r = 0.404, p = 0.022) levels in iNPH before LPS (Fig. 2A). Additionally, we revealed a positive correlation between Aβ42 and APL1β28 levels in iNPH before LPS (r = 0.401, p = 0.023) (Fig. 2B). On the other hand, there were no correlations between Aβ40 and APL1β25, 27, and 28 levels.

**Fig 3:**
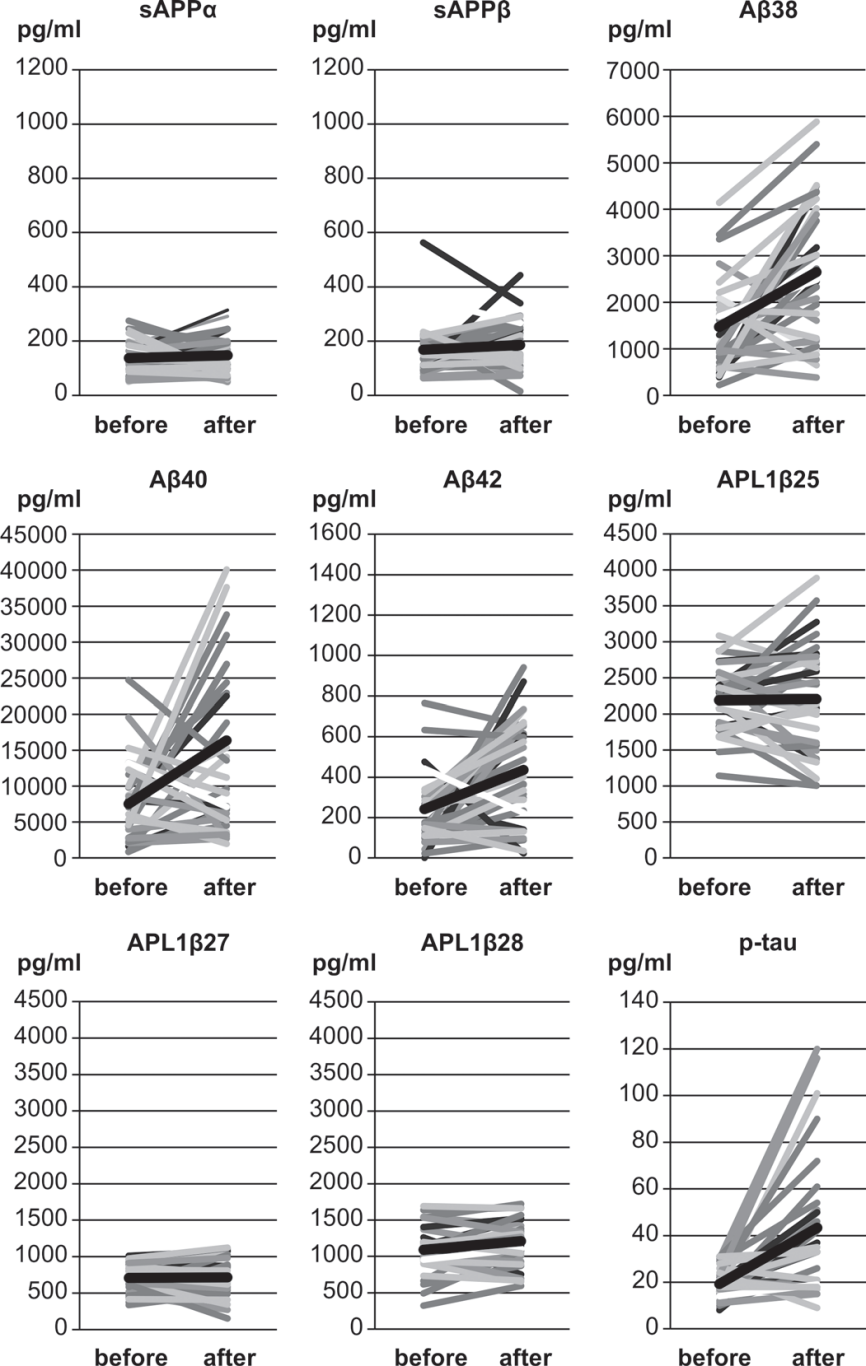
Graph showing the relation between pre- and post-operative lumbar CSF values of individual patients with iNPH for sAPPα, sAPPβ, Aβ 38, 40, and 42, APL1β25, 27, and 28, and p-tau. Abbreviations: CSF, cerebrospinal fluid; iNPH, idiopathic normal pressure hydrocephalus; before, before lumboperitoneal shunt; after, 1 year after lumboperitoneal shunt; sAPP, soluble amyloid precursor protein; Aβ, amyloid β-peptide; APL1β, APLP1-derived Aβ like peptide; p-tau, tau phosphorylated at threonine 181.

Importantly, we found that in patients with iNPH, levels of Aβ38, 40, and 42, as well as tau and p-tau were higher after LPS than before LPS (Table 3; Fig. 3). This was especially true for patients who showed significant improvement after LPS. Among them, increases in Aβ38, Aβ40, and p-tau levels were significant (Aβ38: p = 0.05, Aβ40: p = 0.01: p-tau: p = 0.008). On the other hand, levels of sAPP, sAPPα, sAPPβ, and Aβ43 as well as of APL1β25, 27, and 28 showed no significant differences before and after LPS in both favorable and unfavorable outcome groups.

**Table 3:** Comparison of CSF values in patients with iNPH before and 1 year after LPS.

		Before	After	After/Before	p value
sAPP	**All patients (n = 32)**	478 (244)	510 (250)	1.07	NS
*(ng/mL)*	**Favorable outcome (n = 23)**	480 (259)	546 (261)	1.13	NS
	**Unfavorable outcome (n = 9)**	473 (218)	418 (203)	0.88	NS
sAPPα	**All patients (n = 32)**	137 (64)	147 (70)	1.07	NS
*(ng/mL)*	**Favorable outcome (n = 23)**	130 (64)	164 (72)	1.26	NS
	**Unfavorable outcome (n = 9)**	153 (63)	105 (44)	0.68	NS
sAPPβ	**All patients (n = 32)**	168 (96)	185 (92)	1.10	NS
*(ng/mL)*	**Favorable outcome (n = 23)**	162 (102)	190 (96)	1.17	NS
	**Unfavorable outcome (n = 9)**	185 (84)	174 (86)	0.94	NS
sAPPβ/α	**All patients (n = 32)**	1.32 (0.57)	1.42 (0.80)	1.07	NS
	**Favorable outcome (n = 23)**	1.34 (0.59)	1.37 (0.83)	1.02	NS
	**Unfavorable outcome (n = 9)**	1.27 (0.53)	1.55 (0.75)	1.22	NS
Aβ38	**All patients (n = 32)**	1469 (1007)	2651 (1517)	1.80	<0.001
*(pg/mL)*	**Favorable outcome (n = 23)**	1419 (987)	2756 (1404)	1.94	<0.001
	**Unfavorable outcome (n = 9)**	1599 (1107)	2395 (1831)	1.50	NS
Aβ40	**All patients (n = 32)**	7530 (5581)	16369 (16107)	2.17	0.014
*(pg/ml)*	**Favorable outcome (n = 23)**	7139 (5887)	19224 (17181)	2.69	0.003
	**Unfavorable outcome (n = 9)**	8529 (4883)	9389 (10988)	1.10	NS
Aβ42	**All patients (n = 32)**	241 (195)	435 (258)	1.80	0.001
*(pg/mL)*	**Favorable outcome (n = 23)**	243 (220)	457 (259)	1.88	0.02
	**Unfavorable outcome (n = 9)**	238 (115)	381 (263)	1.60	NS
Aβ43	**All patients (n = 32)**	34.6 (47.0)	35.4 (48.7)	1.02	NS
*(pg/ml)*	**Favorable outcome (n = 23)**	36.9 (52.0)	43.7 (55.6)	1.18	NS
	**Unfavorable outcome (n = 9)**	28.6 (32.4)	15.9 (12.0)	0.56	NS
APL1β25	**All patients (n = 32)**	2188 (444)	2203 (745)	1.01	NS
*(pg/mL)*	**Favorable outcome (n = 23)**	2197 (451)	2209 (762)	1.01	NS
	**Unfavorable outcome (n = 9)**	2162 (454)	2188 (746)	1.01	NS
APL1β27	**All patients (n = 32)**	707 (188)	716 (247)	1.01	NS
*(pg/mL)*	**Favorable outcome (n = 23)**	685 (192)	710 (263)	1.04	NS
	**Unfavorable outcome (n = 9)**	771 (171)	731 (211)	0.95	NS
APL1β28	**All patients (n = 32)**	1091 (318)	1186 (316)	1.09	NS
*(pg/mL)*	**Favorable outcome (n = 23)**	1064 (328)	1194 (334)	1.12	NS
	**Unfavorable outcome (n = 9)**	1162 (296)	1165 (285)	1.00	NS
Tau	**All patients (n = 32)**	112 (92)	186 (116)	1.66	0.004
*(pg/mL)*	**Favorable outcome (n = 23)**	112 (92)	192 (126)	1.72	0.005
	**Unfavorable outcome (n = 9)**	117 (103)	172 (96)	1.47	NS
p-tau	**All patients (n = 32)**	19.2 (6.5)	43.4 (28.9)	2.26	0.001
*(pg/mL)*	**Favorable outcome (n = 23)**	18.2 (6.7)	47.5 (28.9)	2.62	0.001
	**Unfavorable outcome (n = 9)**	21.9 (5.4)	32.8 (32.5)	1.50	NS
Protein	**All patients (n = 32)**	34.4 (9.2)	35.0 (11.1)	1.04	NS
*(mg/dL)*	**Favorable outcome (n = 23)**	33.3 (7.9)	35.1 (11.7)	1.06	NS
	**Unfavorable outcome (n = 9)**	35.2 (11.7)	34.9 (10.1)	0.99	NS
Aβ42/p-tau	**All patients (n = 32)**	19.7 (20.3)	12.1 (7.9)	0.61	NS
	**Favorable outcome (n = 23)**	22.7 (23.2)	11.3 (8.4)	0.50	0.014
	**Unfavorable outcome (n = 9)**	11.9 (5.4)	14.0 (6.7)	1.18	NS

Abbreviations: before, before lumboperitoneal shunt; after, a year after lumboperitoneal shunt; Aβ, amyloid β-peptide; AD, Alzheimer’s disease; APL1β, APLP1-derived Aβ like peptide; CSF, cerebrospinal fluid; iNPH, idiopathic normal pressure hydrocephalus; LPS, lumboperitoneal shunt; NC, normal control; NS, non-significant; p-tau, tau phosphorylated at threonine 181; sAPP, soluble amyloid precursor protein

All *P* values were obtained using the Wilcoxon test. SD values are given in parentheses.

Correlation is significant at the 0.05 level.

**Fig 4:**
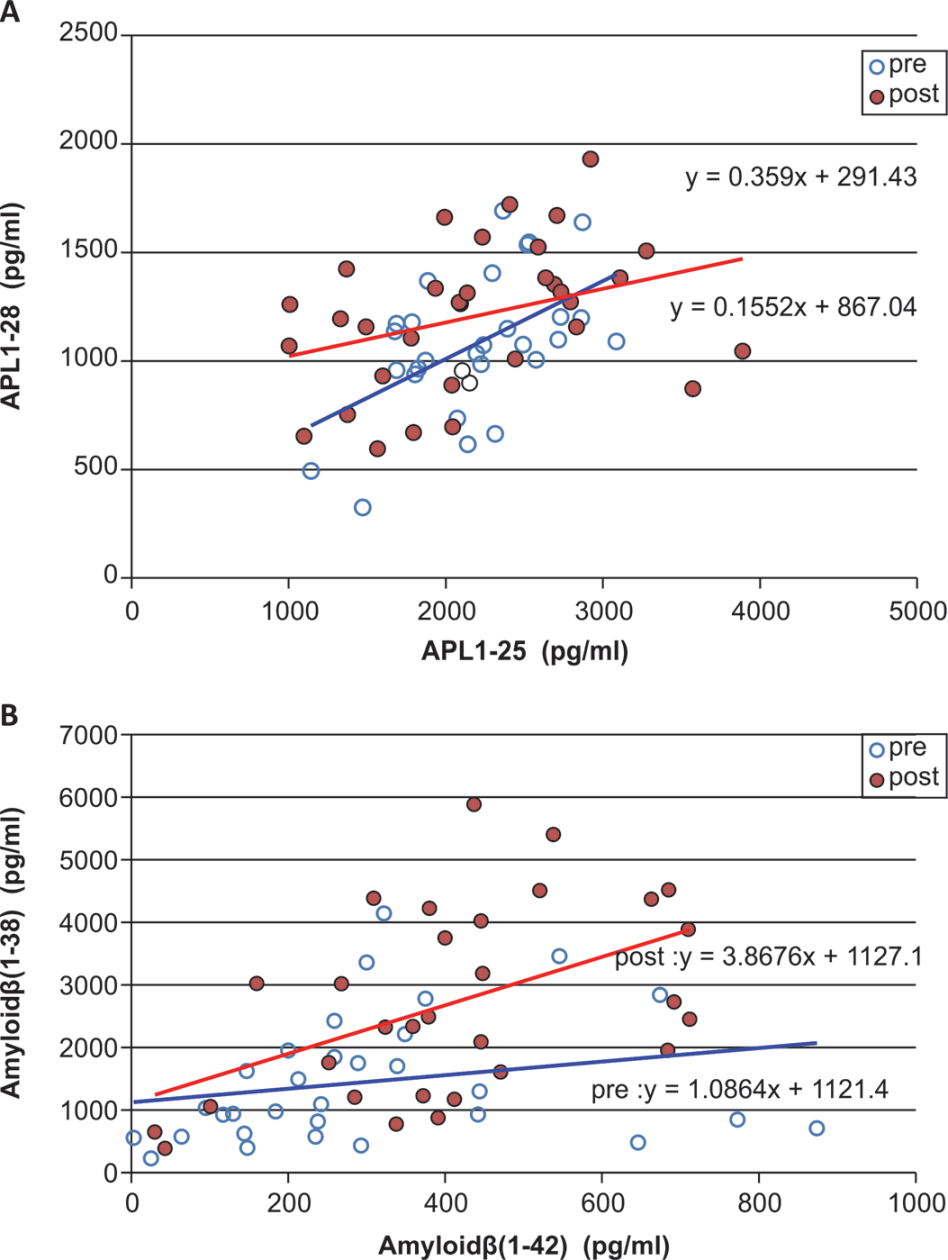
Association of Alzheimer’s disease-related proteins before and after shunting. A: Association between APL1β25 and APL1β28. B: Association between Aβ38 and Aβ42. Abbreviations: pre, before lumboperitoneal shunt; post, after lumboperitoneal shunt; Aβ, amyloid β-peptide; APL1β, APLP1-derived Aβ like peptide.

Levels of sAPPα and sAPPβ showed a positive correlation before and after shunting, with a minor shift from sAPPβ to sAPPα, although not significant. Similarly, APL1β25 and APL1β28, and Aβ38 and Aβ42 also showed a significant positive correlation, with a shift from APL1β28 to APL1β25 and from Aβ42 to Aβ38, respectively, after shunting (Fig. 4).

## Discussion

Consistent with previous reports, we determined that the patients with iNPH showed significantly lower concentrations of sAPPα and sAPPβ in the CSF compared to NCs, suggesting that these could be potential biomarkers for iNPH [8, 23, 24]. Momjian et al. reported that the reduced levels of sAPPα and sAPPβ in iNPH might reflect reduced production of APP-derived proteins, possibly due to reduced brain metabolism in the periventricular zone [25].

In the current study, no significant difference was observed in Aβ42 level between patients with iNPH and NCs in the CSF. On the other hand, Aβ42 level was found to be significantly decreased in patients with AD. This is in partial conflict with previous studies that have frequently reported decreases in Aβ42 levels in the CSF of patients with AD as well as iNPH, compared to NCs [8, 24, 26]. In patients with AD, Aβ42 production is similar to that in NCs, but its clearance is impaired [27]. Furthermore, oligomers of Aβ42 accumulate in the senile plaque and reduce the CSF Aβ42 levels [28]. In contrast, Stenh et al. reported that in patients with iNPH, oligomer formation, favored by altered CSF turnover, could partially mask the antigenic sites on the Aβ42 peptide [29]. As mentioned earlier, APL1β28 is thought to be a surrogate marker for Aβ42 [16, 17]. We found no significant difference in APL1β28 levels between AD, iNPH, and NC, suggesting similar production of Aβ42 in these groups. The absence of a significant difference in Aβ42 levels between iNPH and NCs may be due to the inclusion of individuals with mild cognitive impairment in our NC group.

In the current study, we found that sAPP, sAPPα, sAPPβ, and APL1β25, 27, and 28 levels did not change after LPS. In contrast, levels of Aβ38, 40, and 42 significantly increased after LPS. We also speculated that the shunting would affect α- and β-secretase activities, but found no significant changes in sAPPβ, sAPPα, and sAPPα/β level after LPS, suggesting no effect of shunting on α- or β-secretase activity. Interestingly, we found no change in APL1β28 before and after LPS suggesting that Aβ42 production did not significantly change after LPS. Furthermore, the postoperative shifts from APL1β28 to APL1β25 and from Aβ42 to Aβ38 suggest that the shunting procedure caused a change in γ-secretase activity [30]. More specifically, due to the treatment of iNPH with shunting, Aβ could have been altered to a state less likely to form oligomers or deposit into senile plaques. In many previous reports, Aβ38, 40, and 42 levels have been reported to increase after CSF shunting [8, 31]. Insertion of a shunt reduces the CSF outflow resistance, thereby improving the flow of interstitial-fluid. This seems to inhibit the formation and deposition of oligomers, resulting in the acceleration of Aβ42 discharge into the CSF [32]. The increase in CSF levels of Aβ38, 40, and 42 was especially prominent in the iNPH group with a favorable outcome. In the unfavorable outcome group, the drainage system from the interstitial-fluid space in the brain to the CSF may have been damaged, due to which the CSF level of Aβ would not increase easily, even if LPS did improve CSF outflow resistance. An already damaged interstitial-fluid flow could be the reason why shunting has not been reported to be effective in patients with advanced AD [33].

In the present study, levels of tau and p-tau significantly increased after LPS in the favorable outcome group. Consistent with our findings, increases in tau and p-tau have been reported in lumbar CSF collected after shunting [30, 34, 35]. However, tau has also been reported to decrease in ventricular CSF after ventriculoperitoneal shunting [8, 34]. This discrepancy can be explained by the difference in disease severity, disease duration, and most importantly, the site of CSF collection. In contrast to the favorable outcome group, the unfavorable outcome group did not show significant increases in tau and p-tau levels after LPS. This could be attributed to a similar mechanism described above where interstitial-fluid flow in patients with poor outcome could be compromised. In fact, Aβ oligomers are typically coupled with tau; however, this coupling may be broken when Aβ drainage is facilitated [36]. Thus, improvement in CSF outflow by LPS may facilitate discharge of tau.

To our knowledge, the present study is the first to compare levels of AD-related proteins in the lumbar CSF before and after shunting in patients with iNPH. Importantly, CSF collection during operation through a ventricular puncture may cause contamination of the destructed cerebral parenchyma, thus causing errors in AD-related protein concentrations. It is better to collect lumbar CSF than ventricular CSF before and after operation, because of reduced error and stability of AD-related protein levels in the lumbar method [11, 16]. Moreover, it has been reported that AD-related protein levels change depending on the sites of CSF collection [37, 38]. Our study provides important data by comparing lumbar CSF after a period of 1 year, which is not frequently done in the field.

Our results indirectly show a shift of Aβ42 from oligomer to monomer, and a change in γ-secretase activity due to the improvement in CSF turnover by shunting. Moreover, this procedure could induce increases in CSF levels of Aβ38 and Aβ42 and may delay intracerebral deposition of Aβ, if the drainage system from the interstitial space into the CSF is not completely damaged. It should be noted that a limitation of this study was that the number of patients with iNPH was too small for sufficiently thorough evaluation. Therefore, future studies with increased the number of enrolled subjects are required to confirm our results.
